# Prevalence and Prognosis of Coexisting Frailty and Cognitive Impairment in Patients on Continuous Ambulatory Peritoneal Dialysis

**DOI:** 10.1038/s41598-018-35548-4

**Published:** 2018-11-23

**Authors:** Chunyan Yi, Jianxiong Lin, Peiyi Cao, Jingjing Chen, Ting Zhou, Rui Yang, Shuchao Lu, Xueqing Yu, Xiao Yang

**Affiliations:** Department of Nephrology, The First Affiliated Hospital, Sun Yat-sen University and Key Laboratory of Nephrology, Ministry of Health and Guangdong Province, Guangzhou, China

## Abstract

The aim of this study was to investigate the prevalence of coexisting frailty and cognitive impairment and its association with clinical outcomes in patients on continuous ambulatory peritoneal dialysis (CAPD). Patients on CAPD started to enroll from 2014 to 2016 and ended follow-up by 2017. Frailty was assessed by clinical frailty scale (CFS), and cognitive function was assessed by Montreal Cognitive Assessment (MoCA). Totally 784 CAPD patients were recruited, with median duration of PD 30.7 (8.9~54.3) months. The mean age was 48.8 ± 14.6 years, 320 (40.8%) patients were female and 130 (16.6%) had diabetic nephropathy. Patients with cognitive impairment were more than those with frailty (55.5% vs. 27.6%). Coexisting frailty and cognitive impairment was present in 23.9% patients. Pathway analysis showed that CFS score was negatively associated with MoCA score (β = −0.69, *P* < 0.001). Coexisting frailty and cognitive impairment was associated with decreased patient survival rate (Log-rank = 84.33, *P* < 0.001) and increased peritonitis rate (0.22 vs. 0.11, 0.15 and 0.12 episodes per patient year, respectively; all *P* < 0.001). It was concluded that there was a relatively high prevalence of coexisting frailty and cognitive impairment among patients on CAPD. Frailty was positively associated with cognitive impairment. Coexisting frailty and cognitive impairment increased the risk of adverse outcomes.

## Introduction

Frailty and cognitive impairment are distinct yet frequently overlooked conditions in patients on peritoneal dialysis (PD). These conditions may coexist in patients on PD of any age, not only in older patients. Frailty is a multidimensional syndrome characterized by loss of lean body mass (sarcopenia), weakness and decreased endurance, leading to reduced activity and poor response to stressors^1^. The prevalence of frailty in patients on dialysis has been reported as 25.9~69.4%^2–4^. The most compromised cognitive domains in patients with chronic kidney disease are executive functions, attention, processing speed and memory; domains that are usually assessed in clinical practice using neuropsychological tests^5–7^. Based on different screening instruments, the prevalence of cognitive impairment has been reported to range from 22.3~74.5% in patients on PD^8–10^.

The association between frailty and cognitive impairment has been studied in older populations^11,12^ and patients on hemodialysis (HD)^13–15^, but has not been clearly established in patients on PD. Both frailty and cognitive impairment are barriers to rehabilitation of patients on PD. These two conditions can also lead to lifetime dependency in patients on dialysis, which also in turn is associated with disability, hospitalization and death^3,4,16,17^. Understanding the association between frailty and cognitive impairment among patients on PD may be helpful for early intervention in prevention and management of these conditions. Therefore, this study hypothesized that the prevalence of coexisting frailty and cognitive impairment is common among patients on continuous ambulatory PD (CAPD), and patients with coexisting frailty and cognitive impairment have worse clinical outcomes. This study also investigated the associations between clinical characteristics and frailty and cognitive impairment among patients on CAPD.

## Results

### Demographic and clinical characteristics

**Figure 1:**
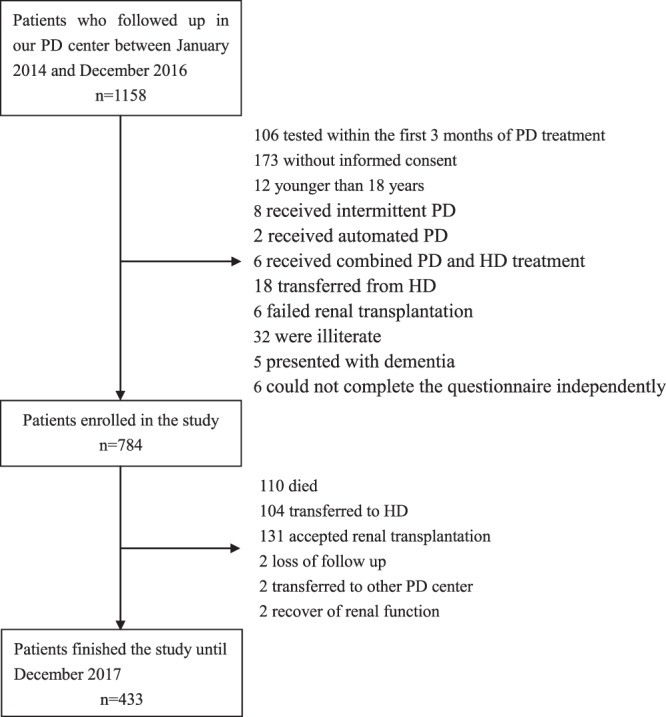
The flow chart shows how patients were selected for the present study.

In total, 1158 patients were assessed for eligibility during the study period. Of these, 106 patients that were tested within the first 3 months of PD treatment, 173 patients that did not provide informed consent and 12 patients that were younger than 18 years were excluded from this study. In addition, eight patients that received intermittent PD, two patients that received automated PD, and six patients that received combined PD and HD treatment were excluded. Finally, we also excluded 18 patients that transferred from HD, six with failed renal transplantation, 32 that were illiterate, five that presented with dementia, and six that could not complete the questionnaire independently. (Fig. 1).

The remaining 784 patients on CAPD were recruited for this study. Demographic and clinical characteristics for these participating patients were shown in Table 1. Participants’ mean age was 48.8 ± 14.6 years and the median duration of PD was 30.7 [interquartile range (IQR) 8.9~54.3] months. In addition, 320 (40.8%) participating patients were female, and 130 (16.6%) had diabetic nephropathy. Frailty was present in 216 (27.6%) patients and cognitive impairment was present in 435 (55.5%) patients; 187 (23.9%) patients had coexisting frailty and cognitive impairment. Compared with the other three groups, patients coexisting frailty and cognitive impairment had higher clinical frailty scale (CFS) score; lower Montreal Cognitive Assessment (MoCA) score, education level and serum creatinine (all *P* < 0.05). Coexisting frailty and cognitive impairment group also had older age, higher proportion of diabetes mellitus and cardiovascular disease, higher level of high-sensitivity C-reactive protein, lower level of serum albumin, serum phosphorus and blood urea nitrogen compared with those patients without frailty or cognitive impairment and with single cognitive impairment (all *P* < 0.05). This group also had higher proportion of female, longer duration of dialysis, higher body mass index and total cholesterol compared with without frailty or cognitive impairment group; had higher level of total cholesterol and measured glomerular filtration rate compared with single frailty group; had higher level of triglycerides compared with single cognitive impairment group (all *P* < 0.05).

**Table 1:** Demographic data and clinical characteristics.

Variables	Total (n = 784)	Without frailty or cognitive impairment (n = 320)	Single frailty (n = 29)	Single cognitive impairment (n = 248)	Coexisting frailty and cognitive impairment (n = 187)	*P* value
MoCA scores (score)	25.0 (21.0~27.0)	27.0 (26.0~28.0)^#^	26.0 (26.0~28.0)^■^	23.0 (20.0~24.0)^▲^	19.0 (16.0~22.0)	<0.001
Frailty classification (n, %)		^#^	■	▲		<0.001
Well (with treated comorbid disease)	474 (60.5%)	287 (89.7%)	0 (0%)	187 (75.4%)	0 (0%)	
Apparently vulnerable	94 (12.0%)	33 (10.3%)	0 (0%)	61 (24.6%)	0 (0%)	
Mildly frail	146 (18.6%)	0 (0%)	25 (86.2%)	0 (0%)	121 (64.7%)	
Moderately frail	62 (7.9%)	0 (0%)	4 (13.8%)	0 (0%)	58 (31.0%)	
Severely frail	8 (1.0%)	0 (0%)	0 (0%)	0 (0%)	8 (4.3%)	
Age (years)	48.8 ± 14.6	39.5 ± 10.3^#^	64.8 ± 8.7	47.0 ± 10.9^▲^	64.7 ± 9.9	<0.001
Gender, female (n, %)	320 (40.8%)	106 (33.1%)^#^	12 (41.4%)	113 (45.6%)	89 (47.6%)	0.003
Education level (n, %)		^#^	■	▲		<0.001
1~6 years	123 (15.7%)	15 (4.7%)	1 (3.4%)	51 (20.6%)	56 (29.9%)	
7~9 years	232 (29.6%)	75 (23.4%)	15 (51.7%)	87 (35.1%)	55 (29.4%)	
10~12 years	215 (27.4%)	77 (24.1%)	6 (20.7%)	83 (33.5%)	49 (26.2%)	
≥13 years	214 (27.3%)	153 (47.8%)	7 (24.1%)	27 (10.9%)	27 (14.4%)	
Primary renal diseases (n, %)						<0.001
Glomerulonephritis	509 (64.9%)	252 (78.8%)	9 (31.0%)	179 (72.2%)	69 (36.9%)	
Diabetic nephropathy	130 (16.6%)	13 (4.1%)	10 (34.5%)	31 (12.5%)	76 (40.6%)	
Renal vascular diseases	64 (8.2%)	18 (5.6%)	6 (20.7%)	17 (6.9%)	23 (12.3%)	
Other	81 (10.3%)	37 (11.6%)	4 (13.8%)	21 (8.5%)	19 (10.2%)	
Diabetes mellitus (n, %)	157 (20.0%)	17 (5.3%)^#^	13 (44.8%)	40 (16.1%)^▲^	87 (46.5%)	<0.001
Cardiovascular disease (n, %)	166 (21.2%)	23 (7.2%)^#^	14 (48.3%)	35 (14.1%)^▲^	94 (50.3%)	<0.001
Duration of dialysis (months)	30.7 (8.9~54.3)	24.5 (8.2~50.6^#^	50.6 (23.7~64.6)	34.9 (8.5~55.5)	33.9 (9.7~58.7)	0.006
Body mass index (kg/m^2^)	22.2 ± 3.3	21.8 ± 3.2^#^	23.3 ± 2.7	22.4 ± 3.3	22.6 ± 3.5	0.004
Hemoglobin (g/L)	111.9 ± 20.1	112.7 ± 20.4	112.7 ± 19.6	111.7 ± 19.9	110.7 ± 20.1	0.74
High-sensitivity C-reactive protein (mg/L)	1.5 (0.6~4.8)	1.0 (0.5~3.0)^#^	3.1 (0.9~7.6)	1.6 (0.6~4.8)^▲^	2.8 (1.1~9.1)	<0.001
Serum albumin (g/L)	36.3 ± 4.4	37.5 ± 4.2^#^	34.7 ± 5.4	36.6 ± 3.9^▲^	34.3 ± 4.4	<0.001
Serum calcium (mmol/L)	2.3 ± 0.2	2.3 ± 0.2	2.3 ± 0.2	2.3 ± 0.2	2.3 ± 0.2	0.26
Serum phosphorus (mmol/L)	1.6 (1.3~2.0)	1.6 (1.3~2.0)^#^	1.6 (1.2~1.9)	1.7 (1.3~2.0)^▲^	1.5 (1.2~1.9)	0.002
Intact parathyroid hormone (pg/ml)	320.4 (157.3~663.5)	319.5 (174.2~583.0)	229.9 (118.1~453.1)	342.5 (165.3~774.9)	315.0 (118.9~587.7)	0.16
Total cholesterol (mmol/L)	4.9 (4.2~5.8)	4.8 (4.2~5.6)^#^	4.4 (3.6~5.3)^■^	5.0 (4.2~5.9)	5.0 (4.2~6.1)	0.03
Triglycerides (mmol/L)	1.5 (1.1~2.1)	1.4 (1.0~1.9)	1.8 (1.1~2.8)	1.5 (1.1~2.1)^▲^	1.7 (1.2~2.4)	0.003
Serum sodium (mmol/L)	138.7 ± 3.1	138.9 ± 2.6	137.9 ± 3.4	138.6 ± 3.2	138.4 ± 3.6	0.22
Blood urea nitrogen (mmol/L)	17.6 (14.3~21.5)	17.8 (14.9~21.1)^#^	17.3 (15.1~19.4)	17.9 (14.4~22.2)^▲^	16.3 (12.8~21.3)	0.06
Serum creatinine (umol/L)	990.5 (760.0~1237.0)	1077.0 (860.0~1334.5)^#^	1024.0 (831.0~1216.5)^■^	1006.5 (799.5~1256.3)^▲^	802.0 (641.0~1020.0)	<0.001
Measured glomerular filtration rate (ml/min/1.73 m^2^)	1.0 (0~3.1)	1.2 (0.1~3.1)	0.1 (0~1.1)^■^	1.0 (0~3.6)	1.0 (0~2.9)	0.003
Clearance index of urea (Kt/V)	2.1 (1.8~2.5)	2.1 (1.8~2.5)	2.0 (1.8~2.25)	2.2 (1.8~2.6)	2.2 (1.9~2.6)	0.07

^#^Comparison between without frailty or cognitive impairment and coexisting frailty and cognitive impairment.

^■^Comparison between single frailty and coexisting frailty and cognitive impairment.

^▲^Comparison between single cognitive impairment and coexisting frailty and cognitive impairment.

MoCA = Montreal Cognitive Assessment.

### Correlations between clinical characteristics and frailty and cognitive function

**Figure 2:**
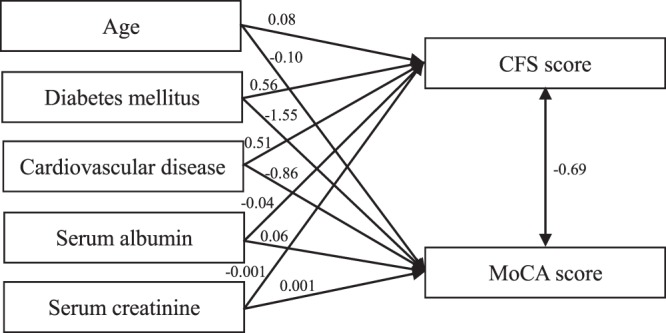
Pathway model of characteristics and frailty and cognitive impairment.

Spearman’s correlation analysis (Table 2) showed that age, duration of dialysis, body mass index, high-sensitivity C-reactive protein and triglycerides were positively correlated with CFS score, while were negatively correlated with MoCA score (all *P* < 0.05). Education level, serum albumin, blood urea nitrogen and serum creatinine were negatively correlated with CFS score, while were positively correlated with MoCA score (all *P* < 0.05). A Mann-Whitney U test (Table 3) showed that those patients who were female, had diabetes mellitus or cardiovascular disease also had higher CFS score and lower MoCA score (all *P* < 0.01). The above variables which were correlated with both CFS score and MoCA score (excluding blood urea nitrogen), hemoglobin and measured glomerular filtration rate were included in the pathway analysis. Among all of the pathway models, age, gender, education level, diabetes mellitus, cardiovascular disease, duration of dialysis, high-sensitivity C-reactive protein, serum albumin, triglycerides, serum creatinine and measured glomerular filtration rate were statistically significantly associated with CFS score and MoCA score (all *P* < 0.05), the association was not significant between other clinical characteristics (e.g. body mass index, hemoglobin) and CFS score or MoCA score. All pathway models showed good model fits. Then, these eleven variables, CFS score and MoCA score were used to conduct a final pathway model. The results showed that CFS score was negatively associated with MoCA score (β = −0.69, *P* < 0.001). Moreover, age, diabetes mellitus and cardiovascular disease were positively associated with CFS score (β = 0.08, *P* < 0.001; β = 0.56, *P* < 0.001; β = 0.51, *P* < 0.001; respectively) whereas serum albumin and serum creatinine were negatively associated with CFS score (β = −0.04, *P* = 0.001; β = −0.001, *P* = 0.01; respectively). Age, diabetes mellitus and cardiovascular disease were negatively related to MoCA score (β = −0.10, *P* < 0.001; β = −1.55, *P* < 0.001; β = −0.86, *P* = 0.004; respectively) whereas serum albumin and serum creatinine were positively associated with MoCA score (β = 0.06, *P* = 0.03; β = 0.001, *P* = 0.03; respectively). (Fig. 2).

**Table 2:** Correlations between clinical characteristics and frailty and cognitive function.

Variables	CFS score		MoCA score	
	r’	*P* value	r’	*P* value
Age (years)	0.71	<0.001	−0.54	<0.001
Education level (years)	−0.24	<0.001	0.43	<0.001
Duration of dialysis (months)	0.17	<0.001	−0.07	0.04
Body mass index (kg/m^2^)	0.13	<0.001	−0.09	0.02
Hemoglobin (g/L)	−0.01	0.69	0.02	0.62
High-sensitivity C-reactive protein (mg/L)	0.27	<0.001	−0.21	<0.001
Serum albumin (g/L)	−0.28	<0.001	0.21	<0.001
Serum calcium (mmol/L)	−0.07	0.04	0.07	0.06
Serum phosphorus (mmol/L)	−0.10	0.008	0.06	0.09
Intact parathyroid hormone (pg/ml)	−0.03	0.47	0.02	0.52
Total cholesterol (mmol/L)	0.01	0.81	−0.09	0.01
Triglycerides (mmol/L)	0.14	<0.001	−0.09	0.01
Serum sodium (mmol/L)	−0.04	0.22	0.06	0.11
Blood urea nitrogen (mmol/L)	−0.08	0.03	0.08	0.02
Serum creatinine (umol/L)	−0.27	<0.001	0.24	<0.001
Measured glomerular filtration rate (ml/min/1.73 m^2^)	−0.12	0.001	0.02	0.66
Clearance index of urea (Kt/V)	0.03	0.42	−0.08	0.05

CFS = clinical frailty scale; MoCA = Montreal Cognitive Assessment; r’ = Spearman’s rank correlation coefficient.

**Table 3:** Comparisons with clinical characteristics and frailty and cognitive function.

Variables	CFS score					*P* value	MoCA score	*P* value
	Well (with treated comorbid disease)	Apparently vulnerable	Mildly frail	Moderately frail	Severely frail			
Gender (n, %)						0.007		0.001
Male	302 (65.1%)	47 (10.1%)	71 (15.3%)	39 (8.4%)	5 (1.1%)		25 (22–27)	
Female	172 (53.8%)	47 (14.7%)	75 (23.4%)	23 (7.2%)	3 (0.9%)		24 (20–26.8)	
Diabetes mellitus (n, %)						<0.001		<0.001
Yes	39 (24.8%)	18 (11.5%)	56 (35.7%)	39 (24.8%)	5 (3.2%)		21 (18–25)	
No	435 (69.4%)	76 (12.1%)	90 (14.4%)	23 (3.7%)	3 (0.5%)		26 (22–27)	
Cardiovascular disease (n, %)						<0.001		<0.001
Yes	37 (22.3%)	21 (12.7%)	68 (41.0%)	36 (21.7%)	4 (2.4%)		21 (18–25)	
No	437 (70.7%)	73 (11.8%)	78 (12.6%)	26 (4.2%)	4 (0.6%)		26 (22–27)	

CFS = clinical frailty scale; MoCA = Montreal Cognitive Assessment.

### Patient survival

**Figure 3:**
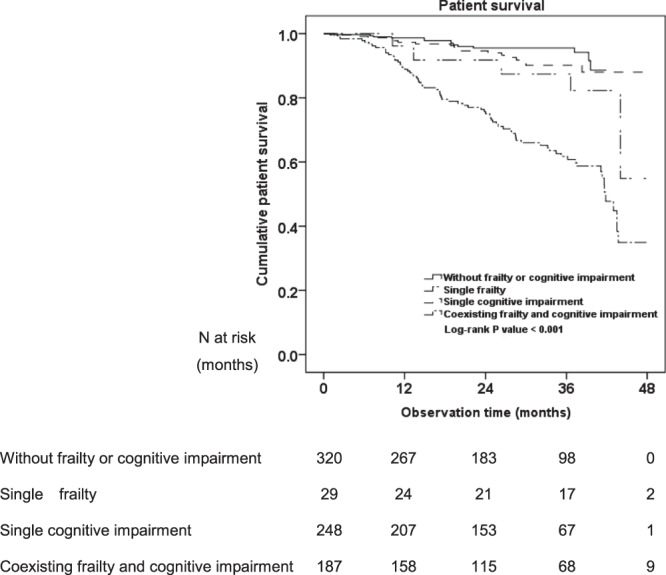
Patient survival.

The median observation time was 28.1 (IQR 15.5~37.2) months. During the study period, 110 (14.0%) patients died, 104 (13.3%) transferred to HD, 131 (16.7%) accepted renal transplantation, two patients were loss of follow up, two (0.3%) transferred to other PD center and two (0.3%) were recover of renal function. Finally, 433 patients finished the study until December 2017. Among 110 deceased patients, 14 patients belonged to without frailty or cognitive impairment group, five belonged to single frailty group, 18 belonged to single cognitive impairment group, and 73 belonged to coexisting frailty and cognitive impairment group. Causes of death were cardiovascular diseases (57.2%), infection (21.8%), tumour (5.5%) and other (15.5%). Results of a Log-rank test showed that patient survival rate in the coexisting frailty and cognitive impairment group was lower than that in the other three groups (Log-rank = 62.47, *P* < 0.001; Log-rank = 5.05, *P* = 0.03; Log-rank = 38.78, *P* < 0.001; respectively) (Fig. 3). Univariate Cox regression analysis showed that age, gender, education level, diabetes mellitus, cardiovascular disease, duration of dialysis, high-sensitivity C-reactive protein, serum albumin, serum sodium, serum creatinine, intact parathyroid hormone, measured glomerular filtration rate, frailty and cognitive function status were associated with mortality in patients on CAPD (all *P* < 0.05). Moreover, coexisting frailty and cognitive impairment [hazard ratios (HR) 2.51; 95% confidence intervals (CI) 1.19~5.29; *P* = 0.02] was associated with increased mortality in patients on CAPD after adjusted demographic data including age, gender, and education level. When we further adjusted other clinical characteristics (e.g. diabetes mellitus, cardiovascular disease, duration of dialysis, high-sensitivity C-reactive protein, serum albumin, serum sodium, serum creatinine, intact parathyroid hormone, measured glomerular filtration rate), the association between coexisting frailty and cognitive impairment and mortality was not significant (HR 0.24; 95% CI 0.73~3.53; *P* = 0.24). (Table 4).

**Table 4:** Risk factors for mortality.

Variants	Univariate Cox regression		Multivariate Cox regression	
	HR (95% CI)	*P* value	HR (95% CI)	*P* value
Age (per year)	1.07 (1.06, 1.09)	<0.001	1.05 (1.02, 1.07)	<0.001
Gender (female)	0.65 (0.43, 0.96)	0.03	0.59 (0.35, 1.002)	0.05
Education level				
1–6 years	1	—	1	—
7–9 years	0.75 (0.46, 1.22)	0.24	1.45 (0.82, 2.54)	0.20
10–12 years	0.52 (0.30, 0.89)	0.02	0.83 (0.44, 1.55)	0.55
≥13 years	0.50 (0.28, 0.90)	0.02	1.15 (0.58, 2.27)	0.68
Diabetes mellitus (yes)	2.94 (2.01, 4.31)	<0.001	1.48 (0.93, 2.35)	0.10
Cardiovascular disease (yes)	3.33 (2.28, 4.87)	<0.001	1.71 (1.09, 2.66)	0.02
Duration of dialysis (per 1 month)	1.01 (1.00, 1.02)	0.003	1.01 (1.00, 1.01)	0.25
High-sensitivity C-reactive protein (per 1 mg/L)	1.02 (1.01, 1.03)	<0.001	1.01 (0.99, 1.02)	0.37
Serum albumin (per 1 g/L)	0.89 (0.86, 0.93)	<0.001	0.98 (0.93, 1.03)	0.39
Serum sodium (per 1 mmol/L)	0.94 (0.89, 1.00)	0.04	0.98 (0.92, 1.05)	0.60
Serum creatinine (per 1 umol/L)	1.00 (0.99, 1.00)	0.002	1.00 (1.00, 1.00)	0.13
Intact parathyroid hormone (per 1 pg/ml)	1.00 (1.00, 1.00)	0.02	1.00 (1.00, 1.00)	0.04
Measured glomerular filtration rate (per 1 ml/min/1.73 m^2^)	0.90 (0.82, 1.00)	0.04	0.86 (0.73, 1.02)	0.08
Frailty and cognitive function status				
Without frailty or cognitive impairment group	1	—	1	—
Single frailty group	0.14 (0.08, 0.24)	<0.001	0.59 (0.19, 1.83)	0.36
Single cognitive impairment group	0.36 (0.15, 0.90)	0.03	1.18 (0.56, 2.53)	0.66
Coexisting frailty and cognitive impairment group	0.22 (0.13, 0.38)	<0.001	1.61 (0.73, 3.53)	0.24

HR = hazard ratios; 95% CI = 95% confidence intervals. Age, gender, education level, diabetes mellitus, cardiovascular disease, duration of dialysis, high-sensitivity C-reactive protein, serum albumin, serum sodium, serum creatinine, intact parathyroid hormone, measured glomerular filtration rate, frailty and cognitive function status were included in this Cox regression analysis.

### Technique survival

**Figure 4:**
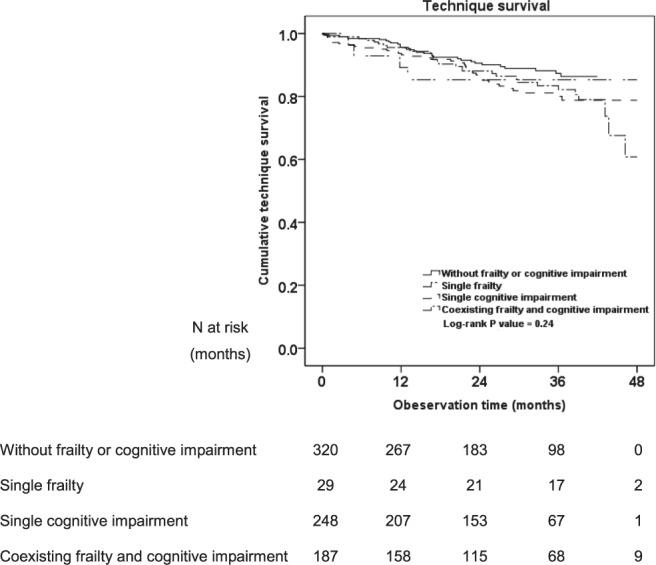
Technique survival.

Among 104 patients who transferred to HD, 31 patients were in without frailty or cognitive impairment group, four were in single frailty group, 39 were in single cognitive impairment group, and 30 were in coexisting frailty and cognitive impairment group. The reasons of technique failure were treatment failure of peritonitis (44.3%), inadequate dialysis (19.2%), ultrafiltration failure (11.5%) and other (25.0%). There were no significant differences on technique survival among the four groups (Log-rank = 4.17, *P* = 0.24) (Fig. 4).

### Peritonitis rate

During the study period, 183 patients accounted for 249 episodes of peritonitis, with the peritonitis rate being 0.15 episodes per patient year. 65 patients coexisting frailty and cognitive impairment accounted for 96 episodes of peritonitis. 58 patients without frailty or cognitive impairment accounted for 77 episodes of peritonitis. Seven patients with single frailty accounted for 11 episodes of peritonitis. 53 patients with single cognitive impairment accounted for 65 episodes of peritonitis. A Poisson analysis showed that the peritonitis rate in the coexisting frailty and cognitive impairment group was higher than that in the other three groups (0.22 vs. 0.11, 0.15 and 0.12 episodes per patient year, respectively; all *P* < 0.001).

## Discussion

This prospective cohort study demonstrated that 23.9% of patients on CAPD presented with coexisting frailty and cognitive impairment. Older age, diabetes mellitus, cardiovascular disease and malnutrition (lower serum albumin and serum creatinine) were associated with both frailty and cognitive impairment among patients on CAPD. Frailty was positively associated with cognitive impairment. Coexisting frailty and cognitive impairment decreased patient survival rate and increased peritonitis rate among patients on CAPD.

Previous studies indicated the prevalence of frailty among patients on dialysis ranges from 25.9~69.4%^2–4^, while that of cognitive impairment ranges from 22.3~74.5%^8–10^. The difference in the prevalence of frailty and cognitive impairment among patients on dialysis may be attributable to different evaluation instruments or different characteristics of study populations. Shimada *et al*.^11^ reported that the prevalence of coexisting frailty and mild cognitive impairment was 2.7% among 4681 community-dwelling older adults in Japan. However, related data for a PD population were absent. Our study involving patients on CAPD showed a much higher prevalence of coexisting frailty and cognitive impairment (23.9%) than that among community-dwelling older adults in the previous study, even though our study population was younger (mean age 48.8 ± 14.6 years). Therefore, the coexistence of frailty and cognitive impairment among patients on PD merits further evaluation.

Similar to general older adult populations, our results showed that older age, diabetes mellitus, cardiovascular disease and malnutrition were associated with both frailty and cognitive impairment among patients on CAPD. The effect of age on frailty and cognitive impairment might be due to age-associated degeneration of frontal lobe structures, which included white matter lesions, loss of gray matter, reduced dendritic branching, and reduced dopaminergic activity^18^. Our results and previous studies found that cardiovascular risk factors including diabetes mellitus and cardiovascular disease were associated with both frailty and cognitive impairment in patients on dialysis^19–23^. Diabetes mellitus was one of risk factors for cardiovascular disease in patients on PD^24^. The literature proposed that the presence of cardiovascular disease in HD patients placed them at an increased risk of subclinical infarcts and white matter disease that might predispose these individuals to cognitive impairment^23^. The underlying mechanism between diabetes mellitus and frailty might be due to accelerating age-related muscle loss^25^. Cardiovascular disease such as atherosclerosis could affect blood flow to the nerves and muscles of the legs, exacerbating sarcopenia, a major component of frailty. Moreover, two studies about brain autopsy showed that the presence of subclinical cardiovascular disease and Alzheimer pathologies was associated with more rapid progression of frailty, and in particular with more rapid decline in walking speed^26^. Previous studies also found that nutrition status was associated with both frailty and cognitive impairment among dialysis patients^19,22,23,27^. Nutrition might influence frailty and cognitive impairment through oxidative stress^28^. Early disease detection and initiation of therapeutic measures for diabetes mellitus, cardiovascular disease and malnutrition might be helpful to decrease the rate of frailty and cognitive impairment among patients on PD.

Our results are the first to provide evidence of the association of frailty and cognitive impairment among patients on PD. This relationship was also confirmed in population on HD^13–15^. Previous studies had found an association between frailty, clinical Alzheimer’s disease (AD) and AD pathology^29–31^. These results might suggest that physical frailty and cognitive impairment share a common underlying pathogenesis. Patients with cognitive impairment might damage areas of brain which were related to motor function (substantia nigra, striatum, major motor cortex and supplementary motor cortex, etc.). The latter included cognitive function areas and motor function areas of brain. Areas of brain such as parietal lobe and hippocampus have dual effects, which not only affect cognitive functions such as memory, but also affect body gait and balance ability. Secondly, patients with cognitive impairment always combined with decreasing motivation to exercise. These factors might be the reasons of why cognitive impairment could lead to frailty. Patients with frailty might reduce exercise. Aerobic exercise contributed to improve cerebral blood flow^32–34^; induce angiogenesis of small-vessel vasculature in the cerebellum, otor cortex, and hippocampus^33,34^; and increase the levels of granulocyte colony-stimulating factor^35^ and brain-derived neurotrophic factor^36^. These changes could improve the cognitive function of patients.

Associations of frailty or cognitive impairment with adverse outcomes in patients on dialysis have been verified in previous studies^3,4,16,17^. However, evidence on the coexistence of frailty and cognitive impairment was lacking. Our results showed that coexisting frailty and cognitive impairment decreased patient survival rate and increased peritonitis rate among patients on CAPD. The possible explanations for our finding are as following. Firstly, frailty and cognitive impairment may be markers of illness and therefore identify patients at greater risk for mortality. Indeed, our results showed that patients with coexisting frailty and cognitive impairment were older, female, less educated, longer duration of dialysis, and more likely to have diabetes mellitus, cardiovascular disease and malnutrition compared with those patients without these two conditions. Secondly, CAPD patients are required to follow complex treatment regimens with frequent dialysis, medication and dietary recommendation changes. Frailty and cognitive impairment can damage executive function which is critical for planning and carrying tasks. The latter can increase the possibility of non-adherence of treatment which may be associated with adverse outcomes^37,38^. Finally, both frailty and cognitive may increase the risk of falls or peritonitis which are also associated with adverse outcomes^10,39,40^.

The main strength of this study was that it was a large-scale single-centre prospective study of patients on CAPD, and incorporated several potential clinical characteristics. Moreover, this is the first study that investigated whether frailty was associated with cognitive impairment in PD patients. However, our study has several limitations. First, a causal association between frailty and cognitive impairment in patients on PD could not be confirmed. Second, we only used the MoCA to assess the cognitive function of patients on PD, and did not combine this with other validated neuropsychological tests. This might have resulted in an underestimation of the prevalence of cognitive impairment.

In conclusion, there was a relatively high prevalence of coexisting frailty and cognitive impairment among patients on CAPD. Frailty was positively associated with cognitive impairment; older age, diabetes mellitus, cardiovascular disease and malnutrition were associated with both frailty and cognitive impairment. Coexisting frailty and cognitive impairment decreased patient survival rate and increased peritonitis rates among patients on CAPD.

## Methods

### Study population

This study was a prospective cohort study. Participants were recruited in a single PD center in Southern China between 1 January 2014 and 31 December 2016. Eligible participants were patients that were aged 18 years or older and had received CAPD treatment for at least 3 months. Participants were excluded if they: had dementia; were transferred from permanent HD; had failed renal transplantation; had received intermittent PD treatment, automated PD treatment or combined PD and HD treatment; or were illiterate, unable to complete the questionnaire independently or unable to provide consent. All patients were followed up until cessation of PD, death, transfer to HD, receiving renal transplantation, loss of follow-up, transfer to another PD center, recover of renal function or study end (31 December 2017). This study was approved by the Human Ethics Committees of Sun Yat-sen University. All participants provided informed consent. All methods in the present study were performed in accordance with the relevant guidelines and regulations.

### Data collection

Demographic data were obtained through medical follow-up files, and included age, gender and education level. Clinical characteristics including primary renal disease, diabetes mellitus, cardiovascular disease, duration of dialysis, body mass index, hemoglobin, high-sensitivity C-reactive protein, serum albumin, serum calcium, serum phosphorus, intact parathyroid hormone, total cholesterol, triglycerides, serum sodium, blood urea nitrogen, serum creatinine, measured glomerular filtration rate and clearance index of urea (Kt/V) were assessed at study enrolment. Diabetes mellitus was defined according to the 2003 American Diabetes Association diagnostic criteria^41^. Cardiovascular disease was diagnosed as myocardial infarction, angina, or history of congestive heart failure, cerebrovascular event and peripheral vascular disease with or without amputation^42^. Kt/V formula was as following. K was renal and peritoneal urea nitrogen removal rate. T was the time. V was calculated using the standardized Watson formula (men V = 2.447 − (0.09156 × age) + (0.1074 × height) + (0.3362 × weight); women V = (0.1096 × height) + (0.2466 × weight) − 2.097). Routine blood, 24 hours urine, and 24 hours peritoneal effluent analysis was used to calculate weekly K values. Finally, we calculated the weekly Kt/V using the result of V obtained by the Watson formula (Kt/V). Clinical events such as peritonitis, death and transfer to HD during the follow-up period were collected.

### Measurement of frailty

Frailty was measured by the CFS, which was demonstrated to have a high intraclass correlation coefficient (0.97) and correlate well with objective measures of frailty (0.80)^43^. The CFS was graded from one to seven: very fit; well without active disease; well with treated comorbid disease; apparently vulnerable; mildly frail; moderately frail; and severely frail. Comorbidity, mobility, ability to cope with activities of daily living and instrumental activities of daily activities were assessed. The reports contained all information required to generate a CFS score for each patient. Frailty was defined as a CFS score more than five.

### Measurement of cognitive function

Cognitive function was measured using the Mandarin version of the MoCA administered by the PD nurses, who had received specialized training for this study. Cognitive domains evaluated in the MoCA included visuospatial and executive functions, name, attention, language, abstraction, delayed recall and orientation^44^. The MoCA Chinese-Language Los Angeles version had a Cronbach’s alpha of 0.78 and 0.79 for Mandarin and Cantonese population, respectively^45^. Total MoCA score was adjusted for education, and higher score represents better cognitive function. Therefore, MoCA score adjusted for education was used in the present study. Cognitive impairment was defined as a MoCA score lower than 26^44,45^.

### Statistical analysis

Descriptive statistics were reported for demographic data and clinical characteristics. Mean ± standard deviation was used to describe normally-distributed continuous variables, median (interquartile range) for skewedly-distributed continuous variables and frequencies (percentages) for categorical variables. Spearman’s correlation analyses and a Mann-Whitney U test were conducted to examine the correlations between clinical characteristics and CFS and MoCA scores. The relationship between clinical characteristics and frailty and cognitive impairment was further conducted by pathway model. Pathway model was tested by robust weighted least squares approach (estimator = WLSMV in Mplus)^46^. Separate pathway models were conducted to examine hypothetical relationships among clinical characteristics, CFS score and MoCA score. Clinical characteristics that were significantly associated with both CFS score and MoCA score were used for the final pathway model. In survival analysis, the exposure factor was frailty and cognitive function status; the primary outcome was death; and the secondary outcomes were technique failure of PD and peritonitis. Death as the end point was used for analysis of patient survival. When calculating death-censored technique survival, the end events included any situation in which a patient on PD switched to HD for more than 3 months. Censored events for both patient and technique survival were renal transplantation, loss to follow-up, transfer to another PD centre, recover of renal function and still on PD at 31 December 2017. In addition, censored data included switching to HD for patient survival and death for technique survival. According to different frailty and cognitive function status, participating patients were divided into four groups: without frailty or cognitive impairment group, single frailty group, single cognitive impairment group and coexisting frailty and cognitive impairment group. Kaplan-Meier curves and Log-rank tests were conducted to compare patient survival and technique survival rate among different groups. Cox regression analysis was used to examine the risk factors for mortality among patients on CAPD. The peritonitis rate was described as episodes per patient year (episodes of peritonitis divided by observation time during the follow-up period) and compared using Poisson analysis. *P* < 0.05 was considered to indicate statistical significance. Pathway models were performed using Mplus Version 7.0 (Muthen & Muthen, Los Angeles, CA). The remaining statistical analyses were performed using IBM SPSS Statistics 19.0 (SPSS Inc., Chicago, IL).

## Electronic supplementary material

Dataset 1

Dataset 2

## Data Availability

The data generated during the study period are available from the corresponding author on reasonable request.
