# Supplementary material for: Prevalence and Prognosis of Coexisting Frailty and Cognitive Impairment in Patients on Continuous Ambulatory Peritoneal Dialysis
Source: Sci Rep. 2018 Nov 23;8:17305. doi: 10.1038/s41598-018-35548-4 (PMC6251896; doi:10.1038/s41598-018-35548-4)
Supplement: Supplementary file 1 — Dataset 1 [file 41598_2018_35548_MOESM1_ESM.pdf]

Prevalence and Prognosis of Coexisting Frailty and Cognitive Impairment in Patients on Continuous Ambulatory Peritoneal Dialysis

Chunyan Yi, Jianxiong Lin, Peiyi Cao, Jingjing Chen, Ting Zhou, Rui Yang, Shuchao Lu, Xueqing Yu, Xiao Yang

Pathway	Estimate	95% CI	Std. Estimate	*SE*	***P* value**
Model 1					
Age → CFS score	0.08	0.08~0.09	0.78	0.004	<0. 001
Age → MoCA score	-0.17	-0.19~-0.15	-0.55	0.01	<0. 001
CFS score ↔ MoCA score	-1.19	-1.46~-0.92	-0.32	0.14	<0. 001
Model 2					
Gender (female) → CFS score	0.20	0.02~0.36	0.10	0.09	0.03
Gender (female) → MoCA score	-1.07	-1.70~-0.44	-0.12	0.32	0.001
CFS score ↔ MoCA score	-2.62	-2.94~-2.30	-0.59	0.16	<0. 001
Model 3					
Education level → CFS score	-0.26	-0.34~-0.18	-0.26	0.04	<0.001
Education level → MoCA score	1.84	1.56~2.11	0.42	0.14	<0.001
CFS score ↔ MoCA score	-2.24	-2.52~-1.96	-0.55	0.14	<0.001
Model 4					
Diabetes mellitus → CFS score	1.17	0.97~1.37	0.43	0.10	<0.001
Diabetes mellitus → MoCA score	-3.85	-4.52~-3.18	-0.34	0.34	<0.001
CFS score ↔ MoCA score	-2.22	-2.52~-1.91	-0.52	0.16	<0.001
Model 5					
Cardiovascular disease→ CFS score	1.18	0.97~1.38	0.43	0.11	<0.001
Cardiovascular disease → MoCA score	-3.44	-4.11~-2.76	-0.31	0.35	<0.001
CFS score ↔ MoCA score	-2.27	-2.57~-1.96	-0.53	0.16	<0.001
Model 6					
Duration of dialysis → CFS score	0.01	0.004~0.01	0.19	0.002	<0.001
Duration of dialysis → MoCA score	-0.01	-0.02~-0.002	-0.08	0.01	0.02
CFS score ↔ MoCA score	-2.64	-2.97~-2.32	-0.59	0.17	<0.001
Model 7					
Body mass index → CFS score	0.04	0.02~0.07	0.13	0.01	0.001
Body mass index → MoCA score	-0.08	-0.18~0.02	-0.06	0.05	0.14
CFS score ↔ MoCA score	-2.66	-2.98~-2.34	-0.59	0.16	<0.001
Model 8					
Hemoglobin → CFS score	-0.001	-0.01~0.003	-0.03	0.002	0.51
Hemoglobin → MoCA score	0.01	-0.01~0.02	0.03	0.01	0.46
CFS score ↔ MoCA score	-2.66	-2.98~-2.33	-0.59	0.17	<0.001
Model 9					
High-sensitivity C-reactive protein → CFS score	0.02	0.01~0.03	0.19	0.004	<0.001
High-sensitivity C-reactive protein → MoCA score	-0.07	-0.11~-0.04	-0.15	0.02	<0.001
CFS score ↔ MoCA score	-2.57	-2.89~-2.25	-0.58	0.16	<0.001
Model 10					
Serum albumin → CFS score	-0.08	-0.10~-0.06	-0.34	0.01	<0.001
Serum albumin → MoCA score	0.24	0.17~0.30	0.23	0.03	<0.001
CFS score ↔ MoCA score	-2.44	-2.76~-2.13	-0.56	0.16	<0.001
Model 11					
Triglycerides → CFS score	0.10	0.06~0.15	0.15	0.02	<0.001

Triglycerides → MoCA score	-0.61	-0.80~-0.42	-0.19	0.10	<0.001
CFS score ↔ MoCA score	-2.75	-3.06~-2.43	-0.60	0.16	<0.001
Model 12					
Serum creatinine → CFS score	-0.001	-0.001~-0.001	-0.33	<0.001	<0.001
Serum creatinine → MoCA score	0.003	0.002~0.004	0.25	0.001	<0.001
CFS score ↔ MoCA score	-2.43	-2.74~-2.11	-0.56	0.16	<0.001
Model 13					
Measured glomerular filtration rate → CFS score	0.000	-0.001~0.000	-0.21	<0.001	<0.001
Measured glomerular filtration rate → MoCA score	0.001	0.000~0.001	0.11	<0.001	0.01
CFS score ↔ MoCA score	-2.61	-2.93~-2.29	-0.58	0.16	<0.001
Final Model					
Age → CFS score	0.08	0.07~0.09	0.60	0.01	<0.001
Gender (female) → CFS score	0.28	0.03~0.53	0.08	0.13	0.03
Education level → CFS score	-0.08	-0.19~0.04	-0.04	0.06	0.21
Diabetes mellitus → CFS score	0.56	0.32~0.80	0.12	0.12	<0.001
Cardiovascular disease → CFS score	0.51	0.28~0.74	0.12	0.12	<0.001
Duration of dialysis → CFS score	0.01	0.002~0.01	0.10	0.002	0.01
High-sensitivity C-reactive protein → CFS score	0.002	-0.01~0.02	0.01	0.01	0.74
Serum albumin → CFS score	-0.04	-0.07~-0.02	-0.10	0.01	0.001
Triglycerides → CFS score	0.001	0.00~0.003	0.05	0.001	0.08
Serum creatinine → CFS score	-0.001	-0.001~0.00	-0.12	<0.001	0.01
Measured glomerular filtration rate → CFS score	-0.06	-0.11~-0.01	-0.10	0.03	0.02
Age → MoCA score	-0.10	-0.13~-0.08	-0.33	0.01	<0.001
Gender (female) → MoCA score	-0.25	-0.85~0.34	-0.03	0.30	0.41
Education level →MoCA score	1.30	1.03~1.57	0.30	0.14	<0.001
Diabetes mellitus → MoCA score	-1.55	-2.15~-0.96	-0.14	0.30	<0.001
Cardiovascular disease → MoCA score	-0.86	-1.46~-0.27	-0.08	0.30	0.004
Duration of dialysis → MoCA score	-0.003	-0.01~0.01	-0.02	0.01	0.57
High-sensitivity C-reactive protein → MoCA score	-0.01	-0.04~0.02	-0.02	0.02	0.58
Serum albumin → MoCA score	0.06	0.01~0.12	0.06	0.03	0.03
Triglycerides → MoCA score	-0.002	-0.01~0.003	-0.04	0.003	0.38
Serum creatinine → MoCA score	0.001	0.00~0.002	0.10	0.001	0.03
Measured glomerular filtration rate → MoCA score	0.08	-0.05~0.21	0.05	0.07	0.23
CFS score ↔ MoCA score	-0.69	-0.96~-0.43	-0.20	0.14	<0.001

CI=confidence interval; Std. Estimate=standardized estimate; *SE*=standard error; CFS=clinical frailty scale; MoCA= Montreal Cognitive Assessment

Final model included age, gender, education level, diabetes mellitus, cardiovascular disease, duration of dialysis, high-sensitivity c-reactive protein, serum albumin, triglycerides, serum creatinine, measured glomerular filtration rate, CFS score and MoCA score.
