# Supplementary material for: Prevalence and Prognosis of Coexisting Frailty and Cognitive Impairment in Patients on Continuous Ambulatory Peritoneal Dialysis
Source: Sci Rep. 2018 Nov 23;8:17305. doi: 10.1038/s41598-018-35548-4 (PMC6251896; doi:10.1038/s41598-018-35548-4)
Supplement: Supplementary file 2 — Dataset 2 [file 41598_2018_35548_MOESM2_ESM.pdf]

Prevalence and Prognosis of Coexisting Frailty and Cognitive Impairment in Patients on Continuous Ambulatory Peritoneal Dialysis

Chunyan Yi, Jianxiong Lin, Peiyi Cao, Jingjing Chen, Ting Zhou, Rui Yang, Shuchao Lu, Xueqing Yu, Xiao Yang

Model	Chi-square	CFI	TLI	RMSEA	WRMR
Reference	the smaller, the better	≥0.95	≥0.95	≤0.08	≤1.00
Model 1	<0.001	1.00	1.00	<0.001	<0.001
Model 2	<0.001	1.00	1.00	<0.001	<0.001
Model 3	<0.001	1.00	1.00	<0.001	<0.001
Model 4	<0.001	1.00	1.00	<0.001	<0.001
Model 5	<0.001	1.00	1.00	<0.001	<0.001
Model 6	<0.001	1.00	1.00	<0.001	<0.001
Model 7	<0.001	1.00	1.00	<0.001	<0.001
Model 8	<0.001	1.00	1.00	<0.001	<0.001
Model 9	<0.001	1.00	1.00	<0.001	<0.001
Model 10	<0.001	1.00	1.00	<0.001	<0.001
Model 11	<0.001	1.00	1.00	<0.001	<0.001
Model 12	<0.001	1.00	1.00	<0.001	0.01
Model 13	<0.001	1.00	1.00	<0.001	<0.001
Final Model	<0.001	1.00	1.00	<0.001	0.001

CFI =comparative fit index; TLI =Tucker-Lewis index; RMSEA= root mean square error of approximation; WRMR =weighted root mean square residual
